# Dose-Response Effects of Exercise and Testosterone Replacement Therapy on Body Composition, Lean Mass, and Heart Rate Responses: A Case Report Using Wearable Technology

**DOI:** 10.7759/cureus.74928

**Published:** 2024-12-01

**Authors:** Gabriel J Sanders, Matthew A Chatlaong, Corey A Peacock

**Affiliations:** 1 Exercise Science, University of Cincinnati, Cincinnati, USA; 2 College of Health Care Sciences, Nova Southeastern University, Fort Lauderdale, USA

**Keywords:** body fat, heart rate zones, muscle mass, sex hormones, wearables

## Abstract

The case report explores the effects of testosterone replacement therapy (TRT) on body composition, lean muscle mass, and fat mass, based on the dosage of TRT and exercise intensity in a 40-year-old male. The purpose of this case study was to evaluate the dose-response relationship of TRT and exercise on muscle hypertrophy and fat loss over an eight-month period, using a validated wrist-worn wearable fitness tracker to measure daily physical activity and heart rate (HR)-based exercise intensity.

The patient, a trained male with 25 years of consistent exercise experience, reported notable declines in strength and increases in body fat despite maintaining a regular workout routine. TRT was prescribed by a physician and self-administered by the patient three times per week, starting at 150 mg per week for the first three weeks and then increasing to 180 mg per week at week four. Daily step counts and strength and aerobic exercise data were tracked utilizing a wrist-worn device (Polar Ignite 2) and an HR chest strap (Polar H10) to monitor time accumulated in five different HR zones (HR Zone 1-5). The study was divided into three phases: pre-TRT (two months of no TRT, just exercise), Phase 1 TRT (three months of TRT and exercise), and Phase 2 TRT (three months of TRT and exercise). Lastly, body composition and basal metabolic rate were assessed via bioelectrical impedance analysis at baseline and throughout the TRT period.

Results indicated a substantial increase in lean muscle mass and a reduction in body fat during the TRT phases. Lean muscle mass increased by 6% during Phase 1 TRT and continued to rise by 3.8% in Phase 2 TRT, while body fat percentage decreased by 1.7% and 1.3% in TRT Phase 1 and TRT Phase 2, respectively. The participant's basal metabolic rate also improved, with a 4.5% increase during Phase 1 TRT and a further 3.2% rise in Phase 2 TRT. The time spent in HR Zones 1-5 significantly (P ≤ 0.007) shifted throughout the study. While time in moderate-to-high-intensity zones (HR Zones 3-5) increased in Phase 1, a trend toward more time accumulated in lower-intensity exercise (HR Zones 1-2) emerged in Phase 2, suggesting a potential adaptation in cardiovascular efficiency. Despite these shifts, overall exercise duration and average and maximal HR responses remained stable across the phases, indicating consistent cardiovascular demand.

Combining TRT with regular aerobic and strength exercise greater than 60 minutes, at least four to five times per week, enhances lean muscle mass and reduces body fat, while the exercise intensity varies from phase to phase. The increase in lean mass was dose-dependent, with larger gains observed early in the TRT supplementation period compared to no TRT. Additionally, the use of wearable technology provided valuable insights into the participant’s HR responses to training. These findings highlight the exercise approaches and HR responses potentially required for significant body recomposition and improved metabolic health with TRT. Further research with larger samples is needed to confirm these results and explore long-term health outcomes.

## Introduction

Testosterone replacement therapy (TRT) with cypionate has gained significant attention in recent years as a potential intervention for increasing muscle mass and reducing fat mass [1]. Cypionate is a synthetic version of the naturally occurring testosterone hormone and is commonly prescribed to treat men with low testosterone levels, a condition known as hypogonadism [2]. With a half-life of approximately eight days, cypionate is typically administered as an intramuscular or subcutaneous injection, and it is the most widely used form of TRT. It is generally available in a 200-mg/mL concentration and supplied in cottonseed oil for extended absorption [2]. The Food and Drug Administration (FDA) recommends administering 50 to 400 mg every two to four weeks, though weekly or even two-to-three-weekly doses are more commonly prescribed in clinical practice to maintain stable testosterone levels [2]. Research suggests that TRT enhances muscle hypertrophy and strength in a dose-dependent manner by increasing protein synthesis and activating other growth-regulating factors [3,4].

Several epidemiological studies have demonstrated a negative correlation between obesity and free testosterone, bioavailable testosterone (free and bound to albumin), and total testosterone (free, bioavailable, and bound to sex hormone-binding globulin) levels [5,6]. This association persists across all age groups, with a significant inverse correlation between total testosterone and obesity, independent of metabolic syndrome [7,8]. The complex interplay between testosterone levels, obesity, and other factors, such as age, diet, and exercise frequency, intensity, and duration, adds further challenges to interpreting TRT’s overall impact on body composition and limits any type of causal explanation. Understanding how these factors interact, especially the exercise intensity required to elucidate muscle mass enhancement, will be essential for evaluating the effectiveness of TRT on muscle gain and fat loss.

Exercise intensity, particularly when measured via heart rate (HR) zone training, plays a crucial role in optimizing fat burning and increasing muscle mass. Research suggests that moderate to high-intensity exercise, typically within 70%-85% of an individual's maximum HR, is effective for burning fat and improving cardiovascular fitness [9]. For muscle hypertrophy, resistance training with adequate intensity and volume, often performed in the 60%-80% of one-repetition maximum (1RM) range, has been shown to be beneficial [10]. While this range offers a good guideline, more specific intensity targets within a narrower 20% range can enhance exercise prescription and help practitioners tailor workouts to maximize lean mass gains and fat reduction. Monitoring HR during exercise allows for fine-tuning workout intensity, ensuring that participants remain within these optimal zones and potentially improving the overall effectiveness of training for both fat loss and muscle gain [11].

Despite promising findings, research on the effectiveness of TRT in response to specific doses of exercise for increasing muscle mass and reducing fat mass remains inconclusive. The variability can be attributed to differences in study design, populations, dosages, natural testosterone concentrations, and TRT duration [3,4]. Additionally, objectively measured daily physical activity and daily exercise intensities are often not reported alongside body composition changes, complicating the assessment of TRT’s combined effects with exercise. Investigating TRT's impact in middle-aged adults (i.e., 40-50 years old) may offer valuable insights, as favorable changes in body composition and muscle mass made earlier in life can improve long-term health outcomes [12].

Therefore, the purpose of this case report is to evaluate the effects of a structured dose of TRT cypionate on muscle mass and fat mass, while also examining how daily physical activity and HR intensity during exercise, tracked via a wrist-worn fitness device, correspond to changes in lean muscle mass and fat loss or gain over a six-month observational period. Given the challenges of controlling extraneous variables in large sample studies, this case report aims to address gaps in the literature by examining the interplay between TRT supplementation, physical activity, and HR-based exercise intensity. By focusing on this interaction and quantifying exercise by time allocated to five different HR zones, the study seeks to provide valuable insights into optimizing body composition through the combined effects of hormone therapy and targeted exercise interventions.

## Case presentation

The patient was a 40-year-old male who had been consistently engaging in strength and aerobic training four to six days per week before starting TRT. He had a 25-year history of regular training but reported notable decreases in strength and increases in body fat over the past 10 years. While the patient did not have a medical diagnosis, his clinical symptoms included extreme fatigue, weight gain, strength, lean mass loss, and upper body fat accumulation despite caloric restriction and regular exercise. He also experienced frequent soft tissue injuries and muscle strains in the gastrocnemius, hamstrings, shoulders, and elbows when exercise intensity increased. The TRT regimen and dosage were prescribed by a board-certified physician as part of the patient's proactive decision to avoid future weight-related diseases. Informed consent was provided by the patient, and the study was approved by the university's institutional review board (University of Cincinnati; approval no.: 2024-0958).

Protocol

Before the beginning of TRT, a complete blood panel and body composition scan with basal metabolic rate assessment were conducted by the physician using bioelectrical impedance (InBody770, Seoul, Korea). Measuring body composition via bioelectrical impedance required the patient to stand barefoot on the scale’s foot sensors while holding the hand sensors for three minutes. All tests were reassessed approximately every three months. The assessment period was divided into three phases that lasted just over eight months, and consistent exercise was maintained between all phases (253 days and 176 exercise sessions). The phase before TRT supplementation (pre-TRT) included no TRT 64 days of observations, which included daily physical activity data and 45 exercise sessions. Then, the TRT supplementation period included two phases, each lasting approximately three months, and the dosing during these phases is stated in the next section, "Doses and Diet." Phase 1 TRT included 91 days of daily physical activity data and 59 exercise sessions. Phase 2 TRT included 98 days of daily physical activity data and 72 exercise sessions. A wrist-worn wearable watch (Polar Ignite 2, Kempele, Finland) was worn daily to quantify step counts, and a chest strap (Polar H10, Kempele, Finland) was worn for each exercise session. The Polar H10 chest strap was linked to the wrist-worn watch to measure HR responses during exercise. Daily physical activity was measured by recording daily step counts. Then for each workout, exercise duration (minutes), HR maximum (beats min⁻¹), HR average (beats min⁻¹), exercise calories expended, and time accumulated in five different HR training zones were measured. A total of three sessions were missed due to technology issues or low battery.

Dose and diet

The patient self-injected all doses with cypionate (200 mg/mL). Throughout the study, all self-injections were administered on Monday, Wednesday, and Friday morning. For the first two weeks, 0.25 ml was administered three times per week, totaling 0.75 mL which equated to 150 mg of cypionate weekly. After the first three weeks, the dose increased to 0.30 mL three times weekly, totaling 0.90 ml which equated to 180 mg of cypionate weekly. This weekly dosage remained at this level for the remainder of the study. The patient made no dietary changes throughout the study (from pre-TRT to the end of Phase 2 TRT). The primary goal was to maintain as much consistency as possible between the pre-TRT period and the TRT supplementation phases.

HR monitoring

HR monitoring was conducted using a validated chest strap device capable of continuously measuring HR during exercise with one-second epochs [13]. The device utilized photoplethysmography technology to detect blood volume changes in the wrist, which were then used to calculate HR. Photoplethysmography is a non-invasive technology that uses light to measure changes in blood volume beneath the skin. The technique involves emitting light into the skin and detecting the amount of light either absorbed or reflected by blood vessels, which varies with the pulsatile flow of blood. The device was worn snugly on the non-dominant wrist (left wrist) to ensure accurate readings according to the manufacturer.

HR zones

For the study, HR data was divided into five distinct zones based on the percentage of the participant's maximum heart rate (HR_max_). Before the pre-TRT phase, the patient completed a graded maximal oxygen consumption (VO_2max_) test on a treadmill using a gas and flow-calibrated metabolic cart (Parvo Medics, TrueOne 2400). When maximal VO_2max_ was achieved, his HR_max_ was 187 beats min⁻¹, thus objectively measuring HR_max_ [14], as opposed to the age-predicted maximum. Each HR zone corresponds to a different level of exercise intensity, with the ranges increasing as a percentage of HR_max_. The five HR zones were calculated based on the patient’s HR_max_ of 187 beats min⁻¹, as follows: Zone 1 (50%-60% of HR_max_) - corresponds to an HR range of 94-111 beats min⁻¹; Zone 2 (60%-70% of HR_max_) - corresponds to an HR range of 112-130 beats min⁻¹; Zone 3 (70%-80% of HR_max_) - corresponds to a HR range of 131-149 beats min⁻¹; Zone 4 (80%-90% of HR_max_) - corresponds to an HR range of 150-167 beats min⁻¹; and Zone 5 (90%-100% of HR_max_) - corresponds to an HR range of 168-187 beats min⁻¹.

The participant was instructed to perform their exercise sessions while the wrist-worn device continuously recorded their HR and to stop recording at the completion of each training session. The data collected was subsequently analyzed to determine the duration and frequency spent in each HR zone. This analysis enabled the researchers to associate the time spent in specific HR zones with changes in lean muscle mass and fat mass, potentially providing insights into the optimal intensity levels for body composition improvements.

Exercise sessions

Based on the patient’s prior exercise history and experience, no formal or structured training program was provided during supplementation. Instead, the patient was encouraged to maintain his usual training routine, which he had followed before starting TRT supplementation. The majority of the patient’s workouts were conducted at a fitness facility; however, when going to the gym was not possible, the patient would complete outdoor runs ranging from two to three miles.

The strength training routine included a combination of strength machines, free weights, dumbbells, and body weight exercises. Each exercise session aimed to include a minimum of 30 minutes of aerobic training and 30 minutes of strength training, ensuring a total exercise duration of at least 60 minutes per session. While many sessions exceeded 60 minutes, the patient consistently adhered to the 60-minute minimum.

The strength training routine was designed to be followed four to six days a week, emphasizing both heavy and light-intensity exercises. Each day targeted specific muscle groups, such as chest and triceps or back and biceps, with varying workloads to promote muscle growth and strength development. For exercises involving heavy lifts, two gradual warm-up sets were completed before the heavy working sets to prepare the muscles and joints for the increased load, reduce injury risk, and enhance performance. The working sets included two to three sets per exercise, with rep ranges of 5-7, equating to 87%-83% of one-rep max (1RM) for heavy lifts to stimulate strength and motor unit activation, and 8-12 reps, equating to 80%-67% 1RM for lighter exercises [15]. Weighted and repetition-based abdominal exercises were also included two to three days per week. Further, throughout Phase 1 TRT, the routine incorporated heavy lifting early in the week, focusing on major muscle groups such as the chest, triceps, back, and biceps, while gradually introducing lighter days toward the end of the week. The routine during Phase 2 TRT was built on this foundation by maintaining a similar heavy and light structure but increased intraday exercise variations (i.e., push-pull variations of chest and back or shoulder push and back pulldowns), with specific attention to compound movements one day each week of deadlifts and squats, performed with lower rep ranges (i.e., four to six repetitions).

Statistical analysis

Descriptive statistics (mean ± standard deviation) were then calculated for all physical activity, exercise, and HR variables recorded by the wrist-worn device. The descriptive statistics provided an overview of the data distribution for each variable across the different phases of TRT. Blood and hormone panels and body composition were reported at the end of each phase but could not be analyzed statistically.

To assess differences in dependent variables, physical activity, exercise duration, exercise calories, and HR variables across the three phases (pre-TRT, Phase 1 TRT, and Phase 2 TRT), multiple mixed-effects models were conducted with time as the fixed factor. The mixed-effects model approach was chosen to account for repeated measures within subjects, providing robust handling of within-subject correlations and variability. Post-hoc pairwise comparisons were performed using the Bonferroni method to control type I errors due to multiple comparisons. Statistical analyses were performed using IBM SPSS Statistics software version 29.0 (IBM Corp., Armonk, NY), and the significance level for all tests was set at P ≤ 0.05. This approach ensured that changes over time were rigorously assessed, accounting for individual variability and repeated measures.

Results

Hormone Panels

**Figure 1:**
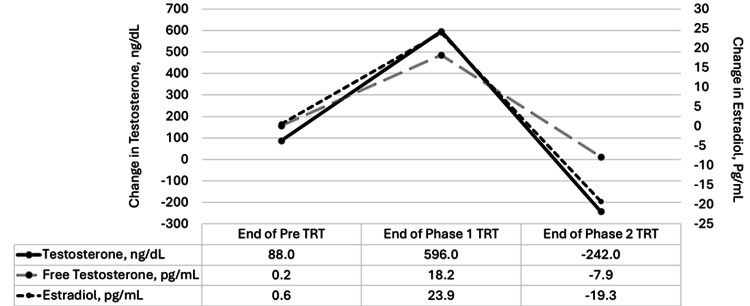
Changes in testosterone, free testosterone, and estradiol levels from baseline to the end of each phase (end of pre-TRT, Phase 1 TRT, and Phase 2 TRT) This data represents the changes from baseline, pre-TRT, and Phase 1 TRT, not actual biological levels. TRT: Testosterone replacement therapy.

Similar to previous research, changes in hormone levels from baseline and the end of each phase are reported, relative to the actual hormone levels at each stage [16]. Throughout the TRT phases, total and free testosterone levels showed significant increases (Figure 1). Both increased modestly from baseline to pre-TTRT. However, by the end of Phase 1 of TRT, testosterone levels increased substantially. By the end of Phase 2 TRT, testosterone levels declined slightly but remained elevated compared to baseline, sustaining enhanced anabolic activity, despite the same TRT dosage. Estradiol, a form of estrogen, followed a similar trend. It increased slightly before treatment and then surged during Phase 1, likely due to the conversion of testosterone into estrogen. By the end of Phase 2 TRT, estradiol decreased but remained higher than baseline, indicating the persistent effects of TRT on estrogen levels.

Body Composition

The body composition results demonstrate noticeable changes across different phases of the study, indicating positive shifts in muscle mass and metabolic rate throughout the TRT phases (Table 1). From baseline to the pre-TRT phase, body weight increased by 1.7%, with small increases of 0.8%, 0.4%, and 0.6% in lean body mass, leg lean mass, and skeletal mass, respectively. Percent body fat initially increased by 0.6% before TRT began, despite regular exercise, and then notably improved throughout the next six months. Body weight continued to increase, gaining an additional 3.8%, but this was largely attributed to a 6% increase in lean body mass and a 6.9% increase in skeletal muscle mass, while body fat percentage decreased by 1.7%.

**Table 1:** Body composition measures and percent changes from each phase TRT: Testosterone replacement therapy.

Body Composition	Baseline	Pre-TRT	%Δ From Baseline	Phase 1 TRT	%Δ From Pre-TRT	Phase 2 TRT	%Δ From Phase 1
Body weight, kg	104.8	106.6	1.7%	110.6	3.8%	113.2	2.3%
Lean body mass, kg	85.6	86.3	0.8%	91.5	6.0%	95.0	3.8%
Leg lean mass, kg	24.9	25.0	0.4%	25.7	3.1%	26.2	1.8%
Skeletal muscle mass, kg	49.0	49.3	0.6%	52.7	6.9%	54.8	3.9%
Percent body fat, %	18.4	19.0	3.3%	17.3	-8.9%	16.0	-7.5%
Basal metabolic rate, kcal	2214	2243	1.3%	2343	4.5%	2419	3.2%
BMI	29.6	30.3	2.4%	31.2	3.0%	32.0	2.6%

By the end of Phase 2 TRT, body weight further increased by 2.3%, accompanied by a 3.8% gain in lean body mass and a 3.9% increase in skeletal muscle mass, indicating a consistent trend of muscle growth. Body fat percentage continued to decline, decreasing by another 1.3%, which signifies a substantial reduction in fat mass. The basal metabolic rate also saw incremental gains throughout the phases, increasing by 1.3% pre-TRT, followed by larger increases of 4.5% and 3.2% during Phase 1 TRT and Phase 2 TRT, respectively. These changes are reflected in the body mass index (BMI), which increased in parallel to the gains in weight and muscle mass. Overall, the data indicates that TRT contributed to increased muscle mass, a higher metabolic rate, and a leaner body composition, despite overall weight gain.

Physical Activity and Heart Rate-Based Exercise Intensity

The patient’s daily step counts showed a marked increase across all phases of the TRT supplementation period (Table 2). Compared to the pre-TRT levels, daily steps increased by 1,823 steps in Phase 1 and by a further 1,819 steps in Phase 2. Exercise duration and exercise calories increased from pre-TRT to Phase 1 TRT with no difference at Phase 2 TRT. This steady progression in physical activity across all phases highlights an overall enhancement in daily movement and exercise capacity associated with TRT despite decreases in biological hormone levels by the end of Phase 2 TRT.

**Table 2:** Average daily data and exercise data from the wrist-worn wearable device Data are means ± SD. ^a ^Significantly different from pre-TRT, P ≤ 0.004 for all. ^b^ Significantly different from Phase 1 TRT, P ≤ 0.007 for all. ^c^ Significantly different from Phase 2 TRT, P ≤ 0.006 for all. TRT: Testosterone replacement therapy.

Physical Activity	Pre-TRT		Phase 1 TRT		Phase 2 TRT	
Exercise variables	Mean ± SD	Max	Mean ± SD	Max	Mean ± SD	Max
Daily step counts	9834 ± 3023^b,c^	15988	11768 ± 4106^a,c^	24326	13206 ± 4141^b^	24484
Exercise time, minutes	62 ± 16	90	71 ± 18	95	66 ± 24	89
HR_max_, BPM	156 ± 20	179	162 ± 14	181	160 ± 14	179
HR_avg_, BPM	122 ± 17	151	126 ± 13	148	122 ± 14	171
Exercise calories expended	677 ± 289^ b^	1298	863 ± 269^a^	1446	766 ± 248	1205

Throughout the three stages of the study, exercise duration, HR_avg_, and HR_max_ remained relatively stable, with no significant differences observed (P ≥ 0.171 for all). These findings suggest that while daily physical activity steps showed marked increases, the cardiovascular demand, as indicated by HR_max_, HR_avg_, and even exercise duration, remained consistent across all phases.

**Figure 2:**
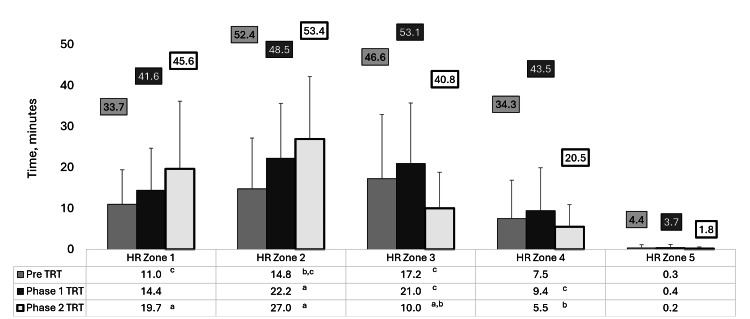
Average (bars) and maximum values (presented in a box above each bar) of exercise time in all five heart rate zones throughout the three phases of the study ^a ^Significantly different from pre-TRT, P ≤ 0.012 for all. ^b ^Significantly different from Phase 1 TRT, P ≤ 0.024 for all. ^c ^Significantly different from Phase 2 TRT, P ≤ 0.028 for all. TRT: Testosterone replacement therapy; HR: Heart rate.

A detailed analysis of HR zones reveals shifting exercise intensities across the study phases (Figure 2). During pre-TRT, the patient predominantly engaged in moderate-intensity activities, spending the most time in the mid-range zones. During Phase 1 TRT, there was an increased time in HR Zones 3 through 5, indicating a shift toward higher-intensity activity. However, Phase 2 TRT showed a significant decrease in the time spent in HR Zones 3-4, with more time accumulated in HR Zones 1-2, suggesting a pivot toward lower-intensity activities. Time in HR Zone 5 remained consistent across all phases, indicating stable but limited high-intensity engagement. In pre-TTRT, the subject accumulated 32 minutes in HR Zones 1-2, while time in HR Zones 3-4 totaled 17.2 minutes, indicating a moderate level of exercise intensity. During Phase 1 TRT, there was a marked increase, with the combined time in HR Zones 3-4 reaching 39.5 minutes, reflecting a higher overall intensity, while HR Zones 1-2 amounted to 22.2 minutes. In Phase 2 TRT, a shift toward lower intensities was observed, with HR Zones 1-2 totaling 36.3 minutes, and HR Zones 3-4 decreasing to 20.0 minutes. These cumulative trends suggest adaptations to varying intensities as a response to TRT.

## Discussion

The case report provides preliminary insights into physiological changes throughout six months of TRT supplementation in a middle-aged male. The cumulative trends in HR intensity suggest adaptations to varying intensities as a response to TRT. Next, the findings demonstrate a robust surge in both total testosterone, free testosterone, and estradiol during Phase 1 TRT, reflecting a strong anabolic response to TRT. This corresponds to an increase of 10% in lean muscle mass with a subsequent 3% decrease in percent body fat over six months of supplementation combined with a high volume and vigorous intensity of exercise. The dramatic rise in estradiol suggests significant aromatization of testosterone into estrogen, a common effect of heightened testosterone. Interestingly, by Phase 2 TRT, both testosterone and estradiol began to decrease, leveling out while still remaining elevated compared to baseline, and muscle mass continued to increase. This suggests that while the body initially responds vigorously to TRT with a sharp hormonal increase, a subsequent plateau or slight reduction occurs as the therapy continues. Exercise appears to resume positive effects despite reductions in hormones. These findings highlight the body's initial rapid hormonal response to TRT, followed by a stabilization phase as hormone levels start to balance out. While the hormone response was stout, data supports a corresponding increase in body weight, lean mass, and a reduction in percent body fat. Although this study is limited to observational case reports, changes may be mediated by hormones, physical activity, and exercise alterations.

The changes from 19% to 16% in body fat composition observed during the study could be partly attributed to improvements in hormone levels, along with notable trends in time spent across five different HR zones. Interestingly, the average time spent in HR Zone 1 and HR Zone 2 increased from pre-TRT (25.8 minutes) to Phase 2 TRT (46.7 minutes), while exercise time in HR Zone 3 and HR Zone 4 was highest during Phase 1 TRT (30.4 minutes). The patient allocated the least time to the most intense HR Zone 5, but no significant changes were observed in HR Zone 5 across the different phases. Despite the patient’s goal of maintaining a similar balance of aerobic and anaerobic training in each session, there are two potential explanations for the shift toward lower-intensity zones (HR Zones 1 and 2) in Phase 2, along with the reduced time in HR Zones 3 and 4. First, the patient’s improved fitness levels throughout months of supplementation and exercise may have led to a blunted HR response, as the improved overall body composition could have made exercise less challenging on the cardiovascular system. Second, the HR response to resistance training might have been reduced due to the increase in strength and lean body mass during Phase 2, even though hormone levels dropped during Phase 2. The findings suggest that when combined with TRT, including aerobic-based exercise and strength training, incorporating both heavier (i.e., 87%-83% 1RM) and lighter (i.e., 80%-67% 1RM) loads may be effective. This could inform future research to investigate optimizing training protocols with TRT. In summary, it appears that the body’s anabolic response can persist despite a shift toward lighter-intensity exercise zones and declining hormone levels, as long as these levels remain elevated compared to non-supplementation periods. This indicates that continuously high levels of TRT may not be necessary to maintain exercise performance improvements.

The body composition changes observed in this study, specifically the increase in lean body mass and the simultaneous decrease in body fat percentage, are consistent with findings from other TRT research [3,4]. In comparison, interventions using glucagon-like peptide-1 (GLP-1) agonists often result in a significant reduction of lean mass, ranging from 40% to 60% of total weight loss [17]. For bariatric procedures, such as gastric sleeve surgery or lap band surgery, studies report a loss of lean mass at 12 months post-surgery of approximately 23.4% of total body weight loss [18]. This highlights a notable difference in the proportion of lean mass lost between pharmacological and surgical interventions [18]. In this case report, the combined effect of TRT and exercise may present a strategy for sustainable weight loss by effectively preserving muscle mass, which is vital for long-term positive health outcomes [19]. It is reasonable to suggest that TRT enhances the effect of exercise and promotes an increase in lean mass while simultaneously reducing body fat percentage, setting it apart from traditional weight loss methods like GLP-1 agonists. This dual effect underscores TRT’s potential effectiveness in body recomposition, making it an advantageous option for preserving muscle mass during fat loss.

A key limitation of this study is its design as a single case report, which restricts the generalizability of the findings to larger populations. Although the study offers detailed and novel insights, being the first to report daily physical activity and HR intensity from eight months of exercise data, it does not distinguish between time accumulated between aerobic exercise and strength training. While quantifying daily strength training volumes in free-living environments is challenging, measuring time spent in five distinct HR zones provides valuable insights into the internal response required for significant lean muscle growth and fat reduction when using exogenous testosterone. It is also possible that despite the patient's exercise and physical activity, patients with a more sedentary history of exercise may have differing impacts of TRT on body composition. However, factors such as individual lifestyle, nutrition, baseline fitness, physical activity levels, and exercise habits significantly influence responses to exercise and TRT, adding complexity and limitations to interpreting the results [20]. These variables can impact the reliability of data assessing internal responses to exercise, and even larger sample studies may face similar limitations in accounting for these influences. Future research should aim to implement similar TRT dosages with standardized training regimens and diets to enhance the comparability of results, though recognizing these controls may be challenging. However, daily assessments of physical activity and quantifying HR-based exercise should be prioritized to provide more comprehensive insights into the effects of TRT on body composition and overall fitness.

## Conclusions

In conclusion, this study provides preliminary findings of the positive effects of TRT combined with aerobic and strength training on body composition over eight months. These effects are reflected by increases in lean body mass, reductions in body fat, and improvements in daily physical activity. The HR data offers valuable insights into a potential dose-response relationship between exercise intensity and TRT, with a shift toward moderate-intensity exercise in Phase 1 TRT followed by lower-intensity activities in Phase 2 TRT. The use of wearable technology to capture real-time data adds a unique dimension to understanding how TRT may influence metabolic health and exercise performance. However, as this is a single case study, further research with larger populations is needed to confirm these findings and their broader implications.
